# Effect of Endurance Exercise Training on Gut Microbiota and ER Stress

**DOI:** 10.3390/ijms251910742

**Published:** 2024-10-05

**Authors:** Eun Ji Yoon, So Rok Lee, Beulah Favour Ortutu, Jong-Oh Kim, Varun Jaiswal, Sooyeon Baek, Su-In Yoon, Sang Ki Lee, Jin Hwan Yoon, Hae-Jeung Lee, Jin Ah Cho

**Affiliations:** 1 Research Center for Microbiome-Brain Disorders, Chungnam University, Daejeon 34134, Republic of Korea; ejyoon224@gmail.com (E.J.Y.); suinyoon@cnu.ac.kr (S.-I.Y.); 2 Department of Food and Nutrition, Chungnam National University, Daejeon 34134, Republic of Korea; sj807sr@cnu.ac.kr (S.R.L.); ortutubeulah@o.cnu.ac.kr (B.F.O.); sooyeonbaek@o.cnu.ac.kr (S.B.); 3 Department of Sport Science, Hannam University, Daejeon 34430, Republic of Korea; jokim1019@hnu.kr (J.-O.K.); yoonjh@hnu.kr (J.H.Y.); 4 Department of Food and Nutrition, Gachon University, Gyeonggi-do 13120, Republic of Korea; varunjais1@gachon.ac.kr; 5 Department of Sport Science, Chungnam National University, Daejeon 34134, Republic of Korea; nicelsk@cnu.ac.kr

**Keywords:** endoplasmic reticulum stress, gut microbiome, inflammation, endurance exercise, tight junction

## Abstract

Regular exercise as part of one’s lifestyle is well-recognized for its beneficial effect on several diseases such as cardiovascular disease and obesity; however, many questions remain unanswered regarding the effects of exercise on the gut environment. This study aimed to investigate the impact of long-term endurance exercise on modulating inflammation and endoplasmic reticulum (ER) stress. Fifteen-week-old male Sprague-Dawley (SD) rats were subjected to six months of endurance treadmill training, while age-matched controls remained sedentary. Results showed that IL-6 mRNA levels in colon tissues were significantly higher in the exercise group compared to the sedentary group. Exercise activated a significant ER stress-induced survival pathway by increasing BiP and phosphorylation of eIF2α (p-eIF2α) expressions in the liver and colon, while decreasing CHOP in the liver. Gene expressions of MUC2, Occludin, and Claudin-2 were increased in the colon of the exercise group, indicating enhanced intestinal integrity. Furthermore, the data showed a positive correlation between microbiota α-diversity and BiP (r = 0.464~0.677, *p* < 0.05). Populations of Desulfovibrio C21 c20 were significantly greater in the exercise group than the sedentary group. Additionally, predicted functions of the gut microbial community in terms of enzymes and pathways supported the enhancement of fatty-acid-related processes by exercise. These findings suggest that prolonged endurance exercise can affect the colon environment, which is likely related to changes in inflammation, ER stress, mucin layers and tight junctions, associated with modifications in the gut microbiome.

## 1. Introduction

Regular physical activity is known to promote physical and mental health [1]. The World Health Organization (WHO) recommends at least 150 min of moderate aerobic activity or 75 min or more of vigorous aerobic activity per week for adults aged 18–64 [2]. However, more than 25 percent of adults worldwide do not get enough physical activity, and excessive sedentary behavior and lack of physical activity are risk factors for various chronic diseases [3].

Exercise enhances immune response, antioxidant capacity, and the efficiency of energy production, reducing the risk of metabolic disorders [1]. Evidence from research has recommended the use of aerobic exercise in the reduction of metabolic syndrome. Specifically, cardiovascular endurance exercises, a subunit under the aerobic category, have been encouraged to enhance metabolic and neuromuscular biogenesis in muscle tissue [4,5]. Although exercise has proven to promote good health, emerging evidence has revealed an association between the intensity of exercise and declining health outcomes [6,7]. Endurance exercise intensity can be classified into low-, moderate-, and high-intensity endurance exercises. Running and swimming are identified as high-intensity endurance exercises, while light jogging and brisk walking are regarded as low- and medium-intensity endurance exercises [8].

Endurance exercises improve physical health and attenuate cellular stress; however, recent investigations have reported the adverse effects of prolonged high-intensity exercises [7,9,10]. High-intensity endurance exercise has shown to induce acute-phase inflammatory response by releasing stress hormones and cytokines such as MCP1, IL-1β, IL-6 and TNFα while decreasing IL-10 levels [11,12]. This can be seen in the prevalence of a gastrointestinal issue called “leaky gut” common to high-intensity endurance athletes. Leaky gut is a condition characterized by the disruption of the protective mucous layer, increased bacterial translocation, and damage to the intestinal epithelium as a result of exercise-induced oxidative stress [13]. However, limited research has focused on the long-term effects of moderate, non-competitive endurance exercise in non-athletes, particularly concerning gut health.

Previous studies have demonstrated that endurance exercises induce the unfolded protein response (UPR) through endoplasmic reticulum (ER) stress, which is caused by elevated reactive oxygen species (ROS) generation during skeletal muscle contraction [14,15]. ER stress occurs when misfolded or unfolded proteins accumulate in the ER as a result of various environmental, physiological, and pathological disturbances in the cells, such as ROS and inflammatory mediators [16]. Binding-immunoglobulin Protein (BiP), a chaperone and central regulator of ER stress, binds to the luminal domains of three ER transmembrane proteins—ER stress sensors—in the resting state of the cells: Inositol Requiring Enzyme 1 (IRE1), Protein kinase RNA-like ER Kinase (PERK), and Activating Transcription Factor 6 (ATF6). BiP dissociates from ER stress sensors as an adaptive response to ER stress and binds to misfolded or unfolded proteins to prevent their further accumulation. This adaptive response triggers a reduction in protein translation, the production of other chaperones and the enhancement of misfolded protein degradation via ER-associated protein degradation (ERAD), therefore suggesting the protective role of ER stress [17].

In addition to influencing ER pathways, endurance exercise may also modulate gut health by altering the composition of the gut microbiota, which plays a vital role in immune regulation and intestinal barrier function [18]. The microbiota of the human gut contains over a thousand bacteria and over three million genes. It influences gut development, intestinal barrier maintenance, host nutritional status, metabolic function, and immune system maturation [19]. The intestinal epithelium consists of a single layer of epithelial cells that are joined securely together. Tight junctions are essential for barrier function. This structure composes complexes of mostly the Claudin and Occludin families, such as Occludin, Zonula Occludin-1 (ZO-1) and Claudin-2, located on the lateral and apical side of the cell membranes, and forms a ring-shaped ribbon around the cells [20]. Recent research suggests that endurance exercise has a positive impact on the stability of tight junctions and the diversity of the gut microbiome. However, the specific mechanisms by which an individual’s exercise performance influences the gut microbiota remain unclear. In addition, there is limited empirical evidence regarding the benefits and underlying mechanisms of prolonged regular endurance exercise training for non-athletes on the gut environment. Therefore, in this work, we aimed to investigate how moderate, long-term endurance exercise influences gut health, particularly by assessing its effects on intestinal inflammation, ER stress, and microbiota composition in non-athletes. Understanding these mechanisms could offer new insights into how regular exercise contributes to gut health and systemic well-being.

## 2. Results

### 2.1. Effect of Endurance Exercise on Inflammatory Response and Intestinal Barrier Protection

We investigated the impact of intense endurance exercise on stress and inflammation in experimental animals. Our exercise protocol was designed to achieve moderate-intensity endurance training, based on previous studies demonstrating the adverse effects of high-intensity endurance exercises [21].

Initially, we assessed systemic stress by measuring serum corticosterone levels and found no difference between the sedentary and exercise groups (Supplementary Figure S1A). Furthermore, superoxide dismutase (SOD) and malondialdehyde (MDA) activities, markers of oxidative stress, showed no significant alterations in the exercise group (Supplementary Figure S1B,C). These findings indicate that moderate-intensity endurance exercise does not induce significant systemic or oxidative stress in the host.

**Figure 1:**
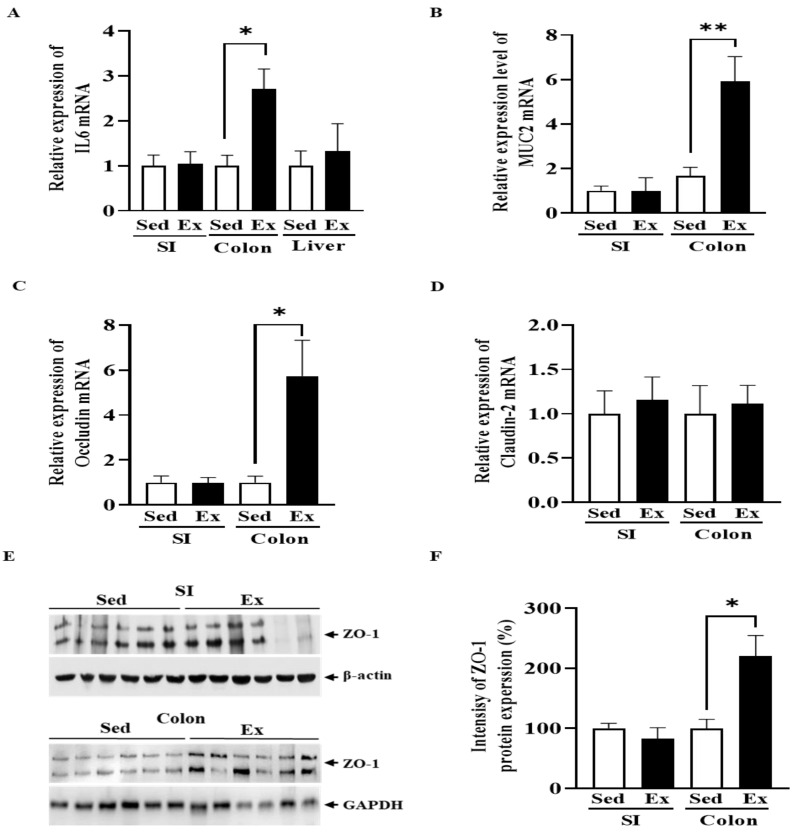
Changes in the inflammatory response, mucin and tight junction of rats by moderate-intensity exercise for six months. The mRNA expression levels of IL6 (**A**), MUC2 (**B**), Occludin (**C**), and Claudin-2 (**D**) in the intestines and liver were measured using real time qRT-PCR. β-actin was used as a loading control. Protein expression of ZO-1 (**E**) in the intestines was measured and quantified using Image J (Version 1.54). n, Sed = 6, Ex = 6. (**F**). β-actin or GAPDH was used as a loading control. Results are presented as mean ± SEM. Significant differences between the exercise and sedentary groups were determined using the Student’s t-test. * *p* < 0.05; ** *p* < 0.01; Sed, sedentary group; Ex, exercise group; SI, small intestine.

Interleukin-6 (IL6), a key pro-inflammatory cytokine known to increase after physical stress, showed a significant increase in both the colon and liver of the exercise group (*p* < 0.05), indicating a localized inflammatory response (Figure 1A).

The intestinal barrier plays an essential role in host defense against pathogens and mucin is the principal biochemical and physical barrier that prevents pathogenic microorganisms contacting intestinal epithelium. To assess the effect of moderate-intensity endurance exercise on intestinal barrier function, we examined the expression of tight junction proteins and glycoproteins responsible for forming a protective barrier in epithelial cells. The mRNA expression of oligomeric mucus gel-forming Mucin 2 (MUC2) was dramatically increased in the colon but not in the small intestine of the exercise group (Figure 1B).

We also evaluated tight junction (TJ) proteins, which play an important role in the epithelial defense barrier. The mRNA expression of Occludin in the colon was significantly higher in the exercise group compared to the sedentary group. Claudin-2 mRNA expression was slightly increased by exercise in both the colon and the small intestine, although this difference was not statistically significant (Figure 1D). In addition, ZO-1, a scaffolding protein that anchors TJ components, showed significantly increased levels in the colon of the exercise group (Figure 1E,F). No significant differences were observed in the small intestine between the sedentary and exercise groups. These results suggest that moderate-intensity endurance exercise may contribute to the reinforcement of the intestinal epithelium, particularly in the colon.

### 2.2. Moderate-Intensity Endurance Exercise Results in Enhanced ER Homeostasis

**Figure 2:**
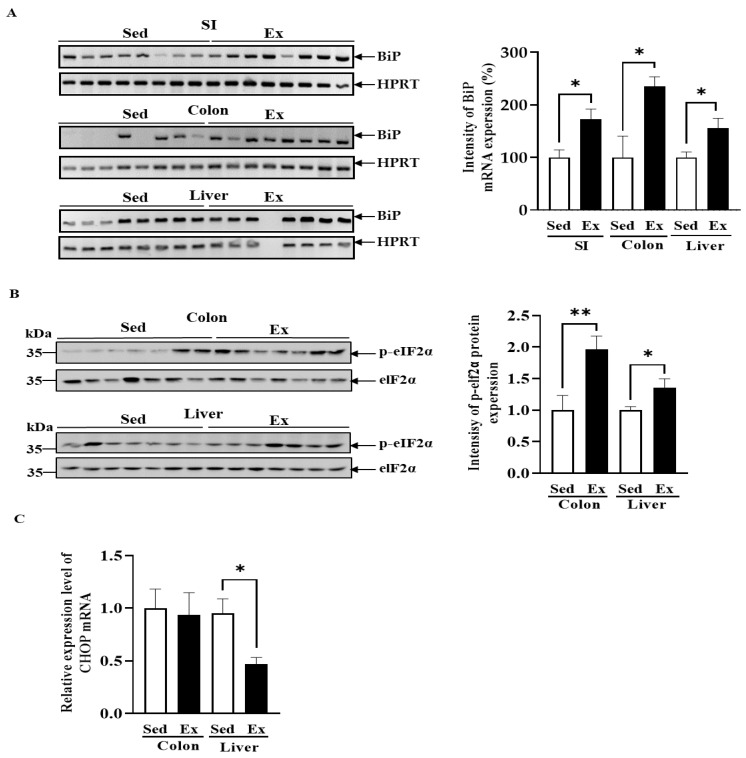
Changes in ER stress by exercise. (**A**) RT-PCR was performed to measure the mRNA expression level of BIP in the intestines and liver of the rats subjected to six months of moderate-intensity exercise. HPRT was used as a loading control. Quantitative mRNA expression levels of BiP were quantified, and results are presented as mean ± SEM. n, Sed = 8, Ex = 8. (**B**) Western blot analysis of p-elF2α in the colon and liver with elF2α, used as a loading control. n, Sed = 7, Ex = 7. (**C**) Real-time qRT-PCR was performed to determine the mRNA expression level of CHOP in the liver and intestines. The significant differences between the exercise and sedentary groups were determined using the Student’s *t*-test. * *p* < 0.05; ** *p* < 0.01; Sed, sedentary group; Ex, exercise group; SI, small intestine.

To investigate whether ER stress occurs during exercise, we examined the expression of several molecular markers of ER stress pathways. The ER chaperone BiP, which possesses anti-apoptotic properties and is a central regulator of ER homeostasis, showed a significant increase in mRNA expression in the exercise group (Figure 2A). This increase in BiP expression reflects an adaptive response to maintain ER homeostasis and prevents cell death under moderate-intensity endurance exercise conditions.

During prolonged ER stress, the PERK pathway becomes activated, leading to the phosphorylation of eIF2α and subsequent inhibition of protein synthesis to prevent further progression toward apoptosis. Our study observed an increase in eIF2α phosphorylation in both the liver and colon tissues of the exercise group (Figure 2B), suggesting that exercise induces ER stress-mediated cell survival mechanisms by inhibiting protein synthesis.

We also assessed the mRNA levels of C/EBP homologous protein (CHOP), a representative ER stress molecule associated with apoptosis. In the liver, CHOP mRNA levels significantly decreased, while no difference was observed in the colon (Figure 2C). All together, these results indicate that the ER stress-induced cell survival pathway was activated by exercise both locally and systemically in the colon and the liver. This suggests that physiological ER stress induced by moderate-intensity endurance exercise may promote adaptation and protect these organs from further stress.

### 2.3. Moderate-Intensity Endurance Exercise Enhances Differential Abundance of Taxa

Our intestinal barrier integrity data highlighted the barrier-strengthening potential of moderate-intensity endurance exercise. To validate these results, we performed a comprehensive analysis of the fecal microbiota from experimental animals to ascertain changes in microbial diversity and bacterial population.

**Figure 3:**
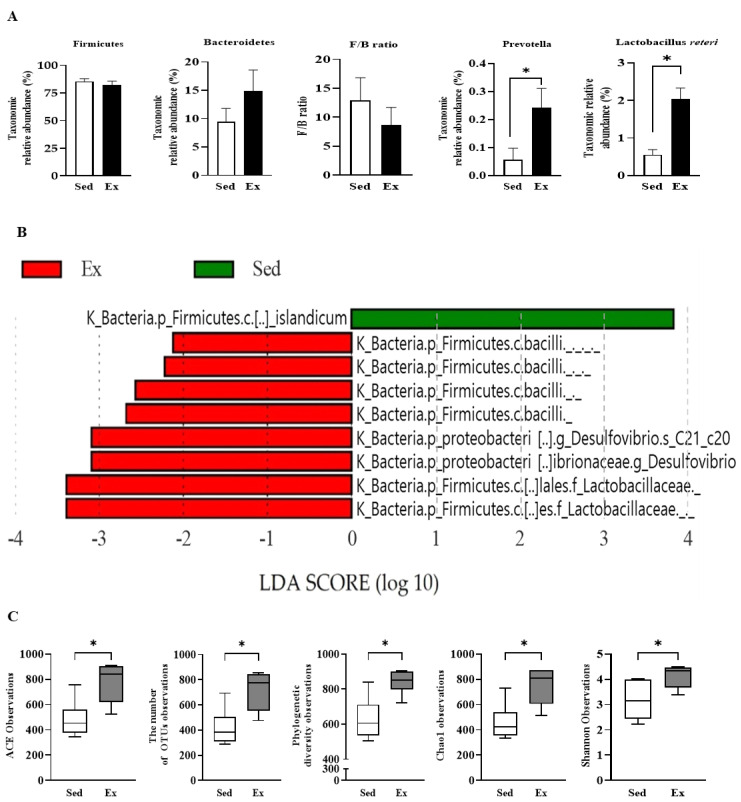
Endurance exercise enhances differential abundance of taxa. (**A**) 16s rRNA sequencing was performed to analyze cecum feces of rats. (**B**) Histogram of LDA scores for differential abundance of bacterial taxa. The cladogram was calculated by LEfSe, and displayed according to effect size. Only taxa meeting an LDA significant threshold of 2 and *p* < 0.05 were shown. LDA, linear discriminate analysis; LEfSe, LDA effect size; k, kingdom; p, phylum; c, class; f, family; g, genes; s, species. (**C**) Microbiome diversity analysis in feces using alpha diversity indices (ACE, OTUs, phylogenetic diversity, Chao, and Shannon). Results are presented as mean ± SEM (Sed n = 10, Ex n = 9); The significant differences between the exercise and sedentary groups were determined using the Student’s *t*-test. * *p* < 0.05; C, sedentary group; E, exercise group.

The populations of Firmicutes were comparable across both groups; however, the Bacteroidetes population was somewhat larger in the exercise group (Figure 3A). Although the Firmicutes to Bacteroidetes (F/B) ratio was higher in the control group, this difference was not statistically significant. Interestingly, Prevotella genera and Lactobacillus reuteri were considerably more abundant in the exercise group.

We employed linear discriminate analysis (LDA) effect size (LEfSe) to obtain the cladogram representation and predominant bacteria of the microbiota in the sedentary and exercise groups (Figure 3B). Our analysis revealed nine taxonomic biomarkers (*p* < 0.05, Kruskal–Wallis test and pairwise Wilcoxon test) with an LDA score (log10 > 2). Of these, one was enriched in the sedentary group, while the other eight were enriched in the exercise group. Notably, Clostridium islandicum was abundant in the sedentary group, a species previously reported to be abundant in chickens with high apparent metabolizable energy level, potentially associated with weight gain [22]. Conversely, Lactobacilaceae species and Desulfovibrio C21 c20 were prevalent in the exercise group.

Group α-diversity of gut microbiota communities, as measured by Abundance-based Coverage Estimators (ACE), Chao1, number of OTUs, Simpson, and phylogenetic diversity indices, varied significantly between groups (Figure 3C). ACE, Chao1, and the number of OTUs were used to assess species richness, while Shannon and phylogenetic diversity indices determined species evenness. Our results showed greater species variety in the exercise group, indicating that continuous moderate-intensity endurance exercise significantly boosted the diversity of the gut microbial community.

### 2.4. Functional Potential of Gut Microbiome and Correlation between Microbial Diversity and ER Stress by Exercise

To predict the functional potential of a community based on amplicon sequence variants (ASVs), we analyzed the Kyoto Encyclopedia of Genes and Genomes (KEGG) orthologs (KO), Enzyme Classification (EC) numbers and MetaCyc pathway abundances [23,24,25]. We identified 11, 40 and 95 entries that were differentially abundant in the sedentary and exercise groups for KO, EC and MetaCyc, respectively (*p* < 0.05, both Welch’s and Wilcoxon rank test).

**Figure 4:**
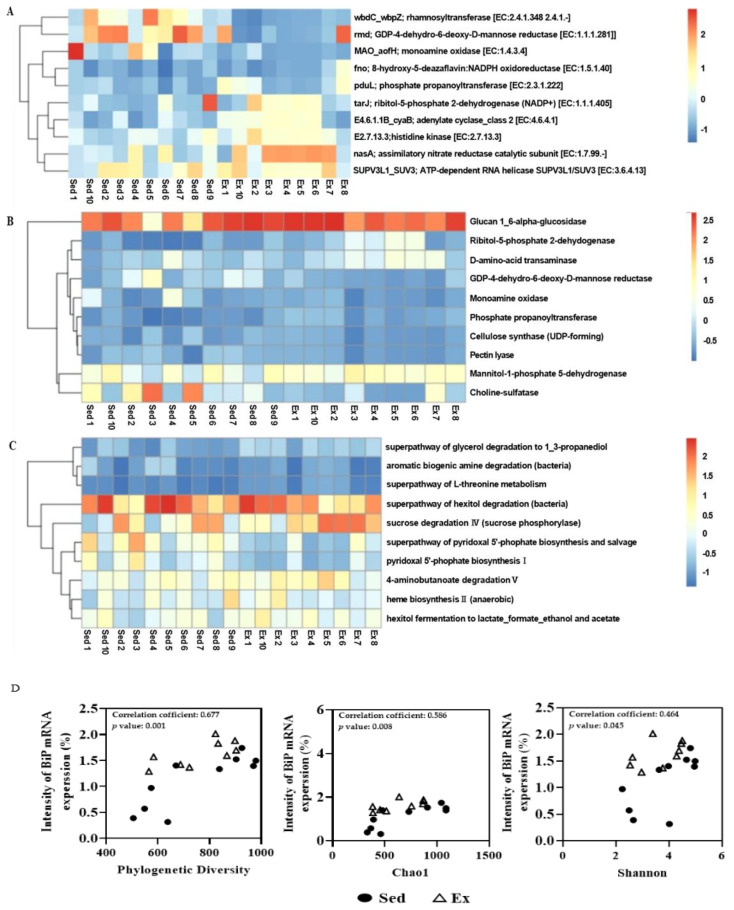
Heatmap and clustering of differential KO (**A**), EC (**B**) and MetaCyc pathway (**C**) abundances (top 10 entries) between sedentary and exercise groups. Correlations between α diversity and ER stress factors. C, sedentary group; E, exercise group. The mRNA expression levels of BiP in colon (**D**) were correlated with various factors of α-diversity. The circle represents the sedentary group, whereas the triangle represents the exercise group.

We further analyzed the differential functional potential using heatmap, plotting the top 10 entries for KO (Figure 4A), EC numbers (Figure 4B) and MetaCyc pathways (Figure 4C) based on the difference within score (median of the largest difference within each group) between the groups.

To elucidate the relationship between microbial diversity and ER stress, we investigate the association between microbial diversity indices and BiP expression in the colon. Our results (Figure 4D) showed that microbial diversity, as measured by phylogenetic diversity, Chao1 and Shannon indices, was induced by moderate-intensity exercise. Importantly, this increased diversity was associated with elevated ER stress in the colon.

These findings collectively suggest that moderate-intensity endurance exercise not only modulates the composition and diversity of the gut microbiome but also influences its functional potential. Moreover, the exercise-induced changes in microbial diversity appear to be intricately linked with ER stress responses in the colon, potentially contributing to the observed improvements in intestinal barrier function and overall gut health.

## 3. Discussion

While several studies have reported a decline in intestinal barrier function, increased inflammation and the redistribution of blood flow during high-intensity endurance exercises leading to intestinal hypoxia and hypoperfusion, less is known about the effect of moderate-intensity endurance exercises [26,27,28]. Our study demonstrated that prolonged moderate-intensity endurance exercise enhances intestinal barrier function, increases ER stress adaptation, and improves gut microbiota diversity, hence providing a strategy to mitigate the harmful effects of high-intensity exercise.

A temporary increase in intestinal inflammation after prolonged moderate-intensity endurance exercise has been associated with strengthening of the immune system to infections and the colonization of commensal microbiota [18]. According to Hernández-Urbán et al. (2024), an increase in the expression of Immunoglobulin A (IgA) as part of the body’s immune response to exercise-induced stress would cause a temporary increase in inflammatory cytokines to protect the gut from potential harm [29]. In this study, moderate-intensity endurance exercise elevated inflammation markers temporarily. This was accompanied by enhanced gut barrier integrity, implying that moderate-intensity endurance exercise protects against gut stress through a coordinated immune response.

While some researchers argue that ER stress disrupts intestinal homeostasis and promotes susceptibility to pathogenic inflammatory response [30], this research revealed that p-eIF2α levels, a key regulatory factor in ER stress, were significantly higher in the colon of the moderate-intensity exercise rat, while BiP expression was significantly elevated in the gut and liver. The elevated p-eIF2α levels reflect a cellular adaptation to enhance protein folding capacity, likely protecting the intestinal epithelium from damage during exercise-induced stress [31]. Concurrently, high BiP levels across various tissues indicate a systemic activation of the ER stress response, mitigating oxidative stress and inflammatory myokine generation [32]. Similar to our findings, a study reported that the deletion of IRE1α from intestinal epithelial cells in mice results in the loss of colonic goblet cells and a reduction in barrier function [17]. Another study emphasized the impact of the deletion of XBP1, resulting in severe intestinal abnormalities and spontaneous intestinal inflammation [33].

ER stress plays an important role in maintaining intestinal epithelial homeostasis. As demonstrated by our findings, it is believed that activating the ER stress pathway to protect the intestinal epithelium of the large intestine can eliminate or reduce the generation of myokines or oxidative stress immediately following endurance exercise.

Goblet cells, characterized as specialized cells of the secretory lineage responsible for producing and secreting mucus, are vulnerable to ER biosynthetic overloading due to the production of large quantities of mucins [34]. This protective mechanism in ER stress highlights the beneficial role of moderate exercise in promoting intestinal health and reducing systemic inflammation and oxidative stress induced by endurance exercise.

The gut microbiota houses the entire population of microorganisms that are colonized in the gastrointestinal tract; therefore, a healthy abundance of these microorganisms promotes metabolic function, antimicrobial function, immunomodulation and integrity of the gut barrier [35]. Several studies have demonstrated that physical activity enhances gut microbiota diversity and promotes beneficial bacterial populations [18,36]. Hence, the higher levels of Bacteroidetes and specific genera, Prevotella and Lactobacillus reuteri in the exercise group of our study, indicate a shift towards a more diverse and possibly more metabolically versatile microbiota. Prevotella is associated with carbohydrate metabolism, which may support increased energy demands and dietary intake during exercise [37], while Lactobacillus reuteri is known for its probiotic properties, contributing to gut health and immune modulation [38]. Furthermore, the increased α-diversity in the exercise group of our study is in agreement with studies that link higher microbial diversity to improved health outcomes [39]. In addition, the absence of a significant difference in the F/B ratio between the study groups was in contrast to studies, suggesting that a lower F/B ratio is associated with better metabolic health. This discrepancy could be due to the moderate-intensity protocol or species-specific microbiota responses, highlighting the need for further investigation into exercise intensity and microbiota dynamics.

In this work, the functional potential of the gut microbiome was also investigated using KO, EC, and MetaCyc pathway analysis. Several of the top 10 differential KO and EC pathways between the exercise and sedentary groups, such as histidine kinase and pectin lyase, are known to promote the production of SCFAs [40,41]. Therefore, microbiome profile changed by exercise could contribute to an increase in SCFAs. Similarly, among the top 10 differential pathways, 2 were recognized as contributing to fatty acid synthesis: hexitol degradation and hexitol fermentation to lactate, formate, ethanol and acetate. Intriguingly, 3 of the top 10 differentially expressed pathways (heme biosynthesis, sucrose degradation, and threonine metabolism) are known to be upregulated in exercise groups [42,43]. Notably, these functional potentials of the gut microbiome are predicted from the 16S rRNA-based amplicon sequences, which may require validation in future studies.

Our study showed an abundance of Desulfovibrio C21 c20 in the exercise group. Although there are few studies which report the function of Desulfovibrio C21 c20, one study showed that the abundance of Desulfovibrio C21 c20 was greater in the gut microbiome of the chow-fed mice group compared to mice on a high-fat diet and was negatively associated with total cholesterol, total triglyceride, body weight, fat mass and blood glucose [44].

Furthermore, exercise-induced microbiota diversity was correlated with reduced inflammation and increased ER stress to maintain a healthier intestinal environment. Taken together, the possible mechanisms from our results would be that exercise induces IRE1β expression and/or changes microbiome profile, including increased Desulfovibrio C21 c20, followed by facilitating lipid metabolism, increasing SCFA and thickening the mucin layer in the colon. Either way, how augmentation in these pathways in the gut microbiome is associated with exercise group would be an important question to pursue in further studies.

In conclusion, the study examined the effect of high-intensity endurance exercise on intestinal inflammation, ER stress and the gut microbiota. The findings suggest that moderate-intensity endurance exercise could be a beneficial intervention to enhance gut health, particularly for non-athletes seeking to improve metabolic and immune function without the risks associated with high-intensity training. Although the present study showed that 6 months of moderate-intensity exercise changed microbiota profiles, our understanding of the effects of exercise on gut microbiota composition and function is limited. Therefore, future research should explore the dose-dependent effects of exercise intensity on gut health, focusing on different durations and intensities to understand their distinct impacts on microbiota composition, inflammation, and ER stress pathways.

## 4. Materials and Methods

### 4.1. Animals

Fifteen-week-old male Sprague-Dawley (SD) rats were purchased from DBL (Eumseong, Republic of Korea), and acclimatized for one week before experiments. The animals were maintained in a controlled environment (23 °C, 45% humidity, 12-h light-dark cycle) and provided a chow diet (DBL, Eumseong, Republic of Korea). Food and water intakes were not restricted. The rats were randomly allocated to two groups: sedentary (Sed, n = 10) or exercise (Ex, n = 9) groups. All experiments were conducted in accordance with protocols approved by the Institutional Animal Care and Use Committee at Hannam University (IRB number: HNU2020-19).

### 4.2. Endurance Exercise Training Protocol

A motorized rodent treadmill (Model DJ-344, Daejong Instrument, Daejeon, Republic of Korea) was used for endurance exercise training. At 0° inclination, the running belt was set horizontally. The rats were trained 3 times per week for 5 min at a running velocity of 5–8 m/min to warm up. The main exercise was performed for 20 min at a running velocity of 15–20 m/min. The exercise ended with a 5-min cool down exercise conducted at 8–10 m/min. The total duration of the training session was 30 min in the first week. From the second week up until the third month, the basal running velocity was increased by 1 m/min on a weekly basis. From the fourth month to the end of the experiment (6 months), the main exercise intensity was performed for 20 min at 20 m/min, 3 times per week. The intensity of exercise corresponded to 65–70% of the maximal oxygen uptake according to previously described protocols with a few modifications [45,46,47]. Twenty-four hours after the training period, the rats were euthanized using CO_2_ to minimize suffering. The liver, ileum and ascending colon were extracted and weighed, and surrounding fat was removed and stored at −80 °C prior to molecular analysis.

### 4.3. RNA Extraction, cDNA Synthesis and Polymerase Chain Reaction (PCR)

Total RNA from liver and gut tissue samples was extracted using TRI reagent (MRC, Cincinnati, OH, USA) in conjunction with the GentleMACS™ Dissociator (Miltenyi Biotec Co., Bergisch Gladbach, Germany). RNA concentration was measured using a Nanodrop spectrophotometer (Thermo Fisher Scientific Inc., Waltham, MA, USA), and cDNA was synthesized using an RT-kit (BioFACT Co., Daejeon, Republic of Korea). Real-time quantitative polymerase chain reaction (RT-qPCR) was conducted using 2X Real-Time PCR Master Mix containing SYBR Green (BioFACT Co. Daejeon, Republic of Korea) on an Agilent real-time qPCR system (Agilent Co., Santa Clara, CA, USA). Primer sequences for real-time qPCR are provided in Table S1. For reverse transcription polymerase chain reaction (RT-PCR), cDNA was combined with 2X Taq Basic PCR Master Mix 2 (BioFACT Co. Daejeon, Republic of Korea), amplified by PCR, and then loaded onto and run on either a 2% agarose gel (for IL6, TNFa, and CHOP) or a 3.5% agarose gel (for XBP1). Gel images were captured under UV light using an AE-9000 E-graph (ATTO, Tokyo, Japan) and quantified using Image J software (NIH Image, Bethesda, MD, USA). Primer sequences for RT-PCR are listed in Table S2.

### 4.4. Enzyme-Linked Immunosorbent Assay (ELISA)

Corticosterone and IL-6 levels in rat serum were measured using a corticosterone parameter assay kit (R&D systems, Minneapolis, MN, USA) and an IL-6 parameter assay kit (R&D systems, Minneapolis, MN, USA). The experiment was carried out according to the manufacturers’ instructions.

### 4.5. Measurement of Lipid Peroxidation

To determine the Malondialdehyde (MDA) level, serum and liver tissue were used. The liver tissue was dissected from each animal (n = 19) and homogenized with phosphate-buffered saline (PBS) containing butylated hydroxytoluene (BHT), and centrifuged at 10,000 *g* for 60 min at 4 °C. Supernatant containing sample solution was added to 20 µM of MDA standard solution. To determine superoxide dismutase (SOD) levels, liver tissue was homogenized in sucrose buffer (0.25 M sucrose, 10 mM Tris, 1 mM EDTA at pH 7.4), and centrifuged at 10,000 *g* for 60 min at 4 °C. Lipid peroxidation (MDA) was determined at 540 nm with a spectrophotometer (Tecan, Grodig, Salzburg, Austria), using the EZ-Lipid peroxidation assay kit (DoGenBio, Seoul, Republic of Korea) and SOD activity was also determined at 450 nm, using the EZ-SOD assay kit (DoGenBio Seoul, Republic of Korea). SOD activity was calculated and expressed as a percentage as follows:

$$SOD\;activity\;(\%)=(1-\frac{{Abs}_{sample}}{{Abs}_{blank}})\times 100$$

### 4.6. Western Blotting

After homogenizing tissues in RIPA buffer using a GentleMACS™ Dissociator (Miltenyi Biotec Co. Miltenyi Biotec Co., Bergisch Gladbach, Germany), protein concentrations were measured using a bicinchoninic acid (BCA) protein assay kit (Thermo Fisher Scientific Inc., Rockford, IL, USA). Protein lysates were dissolved in electrophoresis sample buffer and boiled for 5 min at 100 °C. Proteins were separated in polyacrylamide gels and transferred onto nitrocellulose membranes. Phos-tag gel was used to examine the phosphor form of IRE1α. For Phos-tag gel electrophoresis, Phos-tag reagent (NARD Institute, Ltd., Amagasaki, Japan) and MnCl_2_ were added to the gel to final concentrations of 10 μM and 20 μM, respectively. Membranes were then blocked with 5% skim milk in Twin Tris-buffered saline (TTBS) and incubated overnight at 4 °C with primary antibodies. The primary antibodies used were p-eIF2α (Cell Signaling Technology Danvers, MA, USA), eIF2α (Cell Signaling Technology Danvers, MA, USA), Occludin (Invitrogen Waltram, MA, USA), GAPDH (Invitrogen Waltram, MA, USA), and β-actin (Cell Signaling Technology Danvers, MA, USA). Membranes were then washed with 1× TTBS three times, incubated with secondary goat anti-rabbit antibody (1:10,000, Invitrogen Waltram, MA, USA) or anti-mouse antibody (Invitrogen Waltram, MA, USA) for 1 h, and washed with 1X TTBS three times. Visualization was performed by chemiluminescence Western blot reagents (Thermo Fisher Scientific Inc., Carlsbad, CA, USA) and ChemiDoc system (ATTO Co., Tokyo, Japan).

### 4.7. 16S rRNA Sequencing of Feces

Cecum feces of the rats were stored at −80 °C until required. Total DNA was extracted using the FastDNA^®^ SPIN Kit (MP Biomedicals, Irvine, CA, USA). PCR amplification was performed using fusion primers targeting the V3 to V4 regions of the 16S rRNA gene. For bacterial amplification, fusion primers of 341F (5’-AATGATACGGCGACCACCGAGAT CTACAC-XXXXXXXX-TCGTCGGCAGCGTC-AGATGTGTATAAGAGCAG-CCTACGG GNGGCWGCAG-3’ (the underlined sequence indicates the primer target region)) and 805R (5’-CAAGCAGAAGACGGCATACGAGAT-XXXXXXXX-GTCTCGTGGGCTCGG-AGATGTGTATAAGAGACAG-GACTACHVGGTATCTAATCC-3’) were used. After amplification, the PCR product was confirmed by 1% agarose gel through electrophoresis and visualized using the Gel Doc system (Bio-Rad, Hercules, CA, USA). The amplified product was purified by CleanPCR (CleanNA, Waddinxveen, Netherlands) and evaluated on a Bioanalyzer 2100 (Agilent, Palo Alto, CA, USA) using a DNA 7500 chip. Sequencing was performed by ChunLab Inc. (Seoul, Republic of Korea) using an Illumina MiSeq Sequencing system (Illumina, San Diego, CA, USA) according to the manufacturer’s instructions.

### 4.8. Diversity Analysis

Microbiome diversity analysis was conducted using in-house programs developed by ChunLab Inc. Alpha diversity indices including ACE, Chao, Shannon, Simpson and phylogenetic diversity, rarefaction curves, and rank abundance curves were estimated using EzBioCloud 16S-based MTP (the Chun Lab bioinformatics cloud platform).

### 4.9. Differential Abundance of Taxa

The Quantitative Insights Into Microbial Ecology (QIIME2 version 2021.4) pipeline was utilized to perform ASV-based taxonomic classification analysis of 16S rRNA amplicon sequences [48]. Divisive Amplicon Denoising Algorithm 2 (DADA2) was utilized for denoising, trimming, removal of low-quality reads and filtering of chimeras [49,50]. Taxonomic classification of all ASVs features was performed through sklearn Naïve Bayes taxonomy classifier trained on the Greengenes 13_8 99% OTUs [51]. Finally, differential abundance of taxa was calculated between sedentary and exercise groups according to the linear discriminate analysis effect size (LEfSe) [52]. Level 7, i.e., species level collapsed table converted to biome file format, was utilized for the differential abundance analysis. LEfSe was carried out on galaxy (cloud-based service), which includes formatting, analysis and plotting. Cladogram and bar plots were drawn to visualize the LEfSe results of differentially abundant taxa.

### 4.10. Functional Potential of Bacterial Community with Amplicon Sequences

Prediction of functional composition of metagenome from amplicon sequences in all samples from the sedentary and exercise groups was carried out. PICRUSt2 was selected for the analysis since it is more accurate than the previous method [23]. Quality ASVs obtained from the DADA2 were used for the functional prediction as input in sequences and abundance file in biome format. Kyoto Encyclopedia of Genes and Genomes (KEGG) orthologs (KO), Enzyme Classification numbers (EC) and MetaCyc pathway abundances prediction was carried out through PICRSt2. Further, differential abundances of the functional composition of metagenome in sedentary and exercise groups were studied through ALDeX2, which is based on Welch’s and Wilcoxon rank test [53]. Finally, R was used to visualize the heatmap and clustering of differential KO, EC and MetaCyc pathway abundances through heatmap.

### 4.11. Statistical Analysis

The analysis was performed using SPSS version 25.0 for Windows (SPSS Inc., Chicago, IL, USA). Student’s *t*-test was used to determine the significance of differences in mean values. Correlations between microbial community diversity and various factors were analyzed using Pearson correlation analysis. For statistically significant differences, the *p*-value (*p* < 0.05) was considered, and all data were presented as mean ± standard error of the mean (SEM).

## Data Availability

The original contributions presented in the study are included in the article and Supplementary Materials.

## Supplementary Materials

The following supporting information can be downloaded at: https://www.mdpi.com/article/10.3390/ijms251910742/s1.
