# Supplementary material for: Effect of Endurance Exercise Training on Gut Microbiota and ER Stress
Source: Int J Mol Sci. 2024 Oct 5;25(19):10742. doi: 10.3390/ijms251910742 (PMC11476978; doi:10.3390/ijms251910742)
Supplement: Supplementary file 1 [file ijms-25-10742-s001.zip › ijms-3204193-supplementary.pdf]

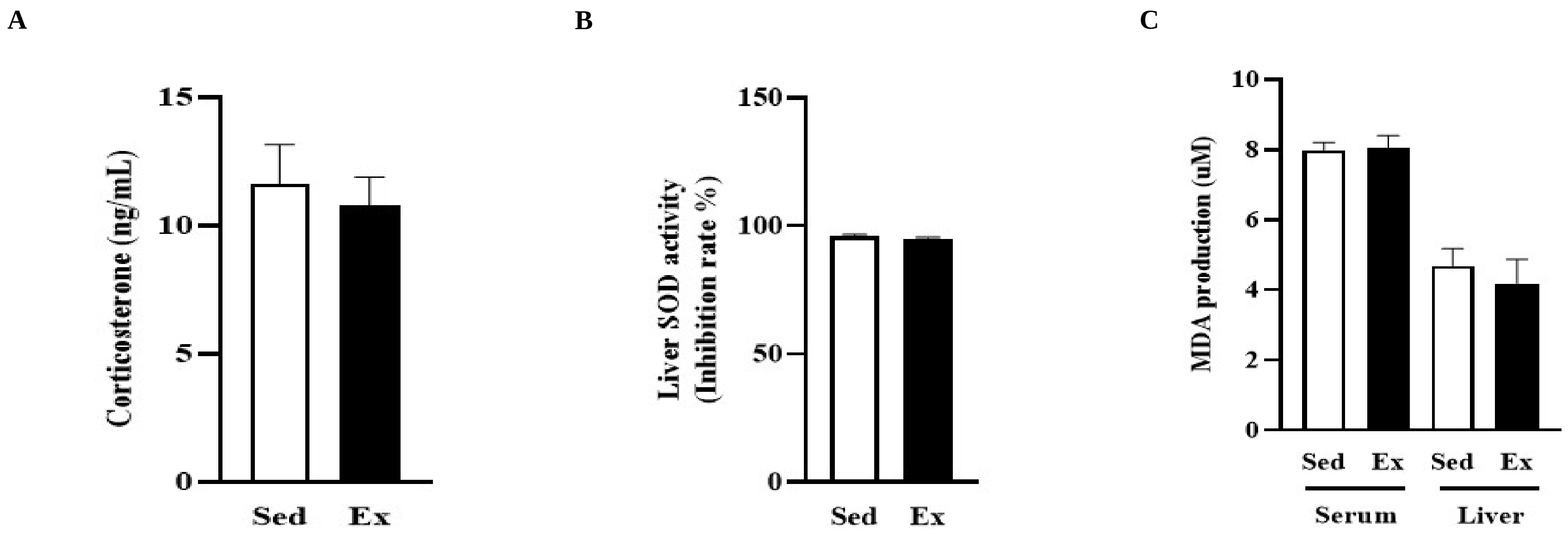

Figure S1. Antioxidant activity and stress levels after moderate-intensity endurance exercise for six months. Corticosterone were measured using ELISA in serum (A). SOD activity in liver was detected (B). The MDA level was measured in serum and liver (D). Results are presented as mean ± SEM (Sed n = 10, Ex n=9). The significant differences between the exercised and sedentary groups were determined using the student's t-test. (*p<0.05, Sed: sedentary group, Ex: exercise group).

Table S1. Real-Time quantitative polymerase chain reaction primers for rats.

Gene	Accession Number	Primer	Sequence (5′→3′)
CHOP	NM_024134.2	Fw	AGC TGG AAG CCT GGT ATG AGG A
		Rv	AGC TAG GGA TGC AGG GTC AA
IL6	NM_012589.2	Fw	TCC TAC CCC AAC TTC CAA TGC TC
		Rv	TTG GAT GGT CTT GGT CCT TAG CC
MUC2	XM_039101270.1	Fw	ACC ACC ATT ACC ACC ACC TCA G
		Rv	CGA TCA CCA CCA TTG CCA CTG
Claudin-2	NM_001106846.2	Fw	TAT GTT GGT GCC AGC ATT GT
		Rv	ACT CCA CCC ACT ACA GCC AC
Occludin	NM_031329.3	Fw	GTT TAC TGG CAG AAC TCG AC
		Rv	CCA GCA TCT GTC TAG GTT TTC
β-actin	NM_031144.3	Fw	TAC CAC CAT GTA CCC AGG CA
		Rv	CTC AGG AGG AGC AAT GAT CTT GAT

Table S2. Reverse transcription polymerase chain reaction primers for rats.

Gene	Accession Number	Primer	Sequence (5′→3′)	Product Length (bp)
BiP	NM_013083.2	Fw	GAT AAT CAG CCC ACC GTA	578
		Rv	GTA TCC TCT TCA CCA GTT GG	
HPRT	NM_012583.2	Fw	GCT GAC CTG CTG GAT TAC AT	110
		Rv	CCC GTT GAC TGG TCA TTA CA	
